# Supplementary material for: Rabies Virus Populations in Humans and Mice Show Minor Inter-Host Variability within Various Central Nervous System Regions and Peripheral Tissues
Source: Viruses. 2022 Nov 28;14(12):2661. doi: 10.3390/v14122661 (PMC9781572; doi:10.3390/v14122661)
Supplement: Supplementary file 1 [file viruses-14-02661-s001.zip › viruses-2008030-supplementary.pdf]

Supplementary tables

Table S1. Ct values of the independent samples selected for viral sequencing. Successfully sequenced samples are indicated in green, non-successful in grey.

Mouse ID	CNS tissues				Non-CNS tissues			
	Brain	Trigeminal ganglion	Spinal cord - dorsal	Spinal cord - ventral	Nuccal skin	Tongue epithelium	Salivary gland	Pharyngeal swab
1							29.82	34.71
2							24.54	27.56
3							26.85	28.97
4							28.4	35.49
5	19.78	21.7	17.68	18.25	32.89	28.28	30.07	
6	23.31	18.19	17.64	19.92	33.12	29.64	29.7	
7	19.14	24.78	16.74	17.33	29.64	31.18	31.38	
8	20.31	20.82	17.59	18.47	30.88	27.45	26.25	
9	21.61	19.12	17.86	17	28.8	23.97	27.42	
10	22.04	19.63	17.22	19.32	33.38	29.59	29.33	

Table S2. Abundance of the observed minor variants (in percentages) detected in the various mice tissues. Yellow cells indicate a SNP.

Mouse ID	Organ	A247C	C2406A	**A5315G (L:G32D)**	**T5898G (L:D226E)**	A6771G	A7704G	G7704A	A8313G	T10668C	C10668T	G10785A	C11031T	T11031C	A11775G	G11775A
2	Pharyngeal swab										>50					
3	Salivary gland			27.3	46.9	41.7										
3	Pharyngeal swab				>50											
5	Trigeminal ganglion															
5	Spinal cord - dorsal															
5	Spinal cord - ventral															
6	Brain															
6	Trigeminal ganglion															
6	Spinal cord - dorsal	25.2														
6	Spinal cord - ventral	29.7														
6	Nuchal skin								24.7							
7	Brain															
7	Trigeminal ganglion		21.0													
7	Spinal cord - dorsal															

7	Spinal cord - ventral															
7	Nuchal skin															
8	Brain										41.3					
8	Trigeminal ganglion									29.1	>50					
8	Spinal cord - dorsal										44.7					
8	Spinal cord - ventral										36.5					
8	Nuchal skin										21.9					
8	Tongue epithelium									33.2	>50					
8	Salivary gland															
9	Brain															
9	Trigeminal ganglion															
9	Spinal cord - dorsal															
9	Spinal cord - ventral															
9	Nuchal skin															
9	Tongue epithelium															
9	Salivary gland															
10	Brain						27.2						26.7			20.5
10	Trigeminal ganglion						35.5					34.4	27.4			37.1
10	Spinal cord - dorsal						>50	36.5					>50	39.5	32.4	>50
10	Spinal cord - ventral						>50	38.0					>50	34.8	37.0	>50
